# Transcriptome Regulation Mechanisms Difference between Female and Male *Buchloe dactyloides* in Response to Drought Stress and Rehydration

**DOI:** 10.3390/ijms25179653

**Published:** 2024-09-06

**Authors:** Muye Liu, Yalan Su, Ke Teng, Xifeng Fan, Yueseng Yue, Guozeng Xiao, Lingyun Liu

**Affiliations:** 1 The College of Horticulture and Garden, Yangtze University, Jingzhou 434052, China; lmy6511999@163.com (M.L.); suyalan18@163.com (Y.S.); 2 Institute of Grassland, Flowers and Ecology, Beijing Academy of Agriculture and Forestry Sciences, Beijing 100097, China; tengke.123@163.com (K.T.); fanxifengcau@163.com (X.F.); yysen2008@sina.com (Y.Y.)

**Keywords:** drought stress, *Buchloe dactyloides*, dioecious, transcriptome, WGCNA

## Abstract

Drought, a pervasive global challenge, significantly hampers plant growth and crop yields, with drought stress being a primary inhibitor. Among resilient species, *Buchloe dactyloides*, a warm-season and dioecious turfgrass, stands out for its strong drought resistance and minimal maintenance requirements, making it a favored choice in ecological management and landscaping. However, there is limited research on the physiological and molecular differences in drought resistance between male and female *B. dactyloides.* To decipher the transcriptional regulation dynamics of these sexes in response to drought, RNA-sequencing analysis was conducted using the ‘Texoka’ cultivar as a model. A 14-day natural drought treatment, followed by a 7-day rewatering period, was applied. Notably, distinct physiological responses emerged between genders during and post-drought, accompanied by a more pronounced differential expression of genes (DEGs) in females compared to males. Further, KEGG and GO enrichment analysis revealed different DEGs enrichment pathways of *B. dactyloides* in response to drought stress. Analysis of the biosynthesis and signaling transduction pathways showed that drought stress significantly enhanced the biosynthesis and signaling pathway of ABA in both female and male *B. dactyloides* plants, contrasting with the suppression of IAA and JA pathways. Also, we discovered *BdMPK8-like* as a potential enhancer of drought tolerance in yeast, highlighting novel mechanisms. This study demonstrated the physiological and molecular mechanisms differences between male and female *B. dactyloides* in response to drought stress, providing a theoretical basis for the corresponding application of female and male *B. dactyloides.* Additionally, it enriches our understanding of drought resistance mechanisms in dioecious plants, opening avenues for future research and genetic improvement.

## 1. Introduction

Drought is a widespread global phenomenon [1], with severity influenced by factors like rainfall frequency, evaporation rates, and soil moisture levels [2]. Plants have evolved various mechanisms for adapting to arid environments over the long term. These strategies include drought escape, avoidance, tolerance, and recovery, which collectively affect their overall performance during drought and rewatering [3].

Research into plant drought resistance has entered an era of integrating physiological and multi-omics analyses. DNA sequencing technologies, such as Next Generation Sequencing (NGS) and PacBio Single Molecule Real-Time (SMRT) sequencing, play pivotal roles in uncovering the molecular mechanisms underlying plant responses to drought stress. SMRT sequencing, in particular, offers extensive transcript sequence data while reducing assembly errors, making it advantageous for species with non-reference or low-quality reference genomes [4], Next Generation Sequencing (NGS) determines DNA sequences by capturing terminal markers of newly synthesized bases. It enables high-throughput sequencing of both DNA and RNA samples [5]. For instance, researchers employed transcriptomic analysis to explore the underlying mechanisms in sunflower leaves during drought stress and subsequent rewatering, identifying genes that respond positively or negatively to these conditions [6]. Transcriptomic analysis revealed that COR significantly enhances drought resistance in *Carex breviculmis*. This study provides a new perspective on the functional analysis of genes involved in the COR-mediated signaling pathway [7]. Transcriptomic analysis of *Astragalus membranaceus* under drought stress revealed insights into the molecular mechanisms. It identified that plant hormone signaling pathways and related transcription factors play crucial roles in *A. membranaceus* response to drought stress [8].

Current research on plant drought resistance predominantly centers on monoecious plants, with limited exploration of the molecular regulatory mechanisms between genders in dioecious plants under drought stress. While sexual dimorphism is common in animals, it is less frequently observed in plants [9], about 65% of reported species in seed plants are dioecious [10], but most angiosperms are hermaphroditic, with only 6% exhibiting dioecism [11]. The origin of dioecious plants is often associated with evolutionary differences between sexes, primarily excluding differences in sexual organ characteristics [12]. Due to ecological, morphological, and physiological differences between sexes, dioecious plants reflect adaptations of genders to varying resource demands [13]. Non-biological environmental stresses may contribute to the evolution of dioecious plants [14], with such differences leading to the specialization of a single sex during evolution, resulting in sex-specific traits. Studies indicate a gender bias in over half of dioecious plants [15]. Gender bias favoring male growth and development is widespread in trees and grapevines. For example, in Populus, male plants exhibit stronger antioxidative properties than females under drought stress [16]. While *Pistacia lentiscus* females show lower CO_2_ assimilation rates and stomatal conductance than males under drought stress [17]. Under drought conditions, the male plants of Populus have a higher net photosynthetic rate and carboxylation rate [18].

Buffalo grass (*Buchle dactyloides*) is extensively cultivated in China for applications such as airports and slope protection, prized for its smooth appearance, strong stress resistance, and ability to thrive in poor soils [19]. As one of the few dioecious grass species used for lawns, *B. dactyloides* exhibits physiological differences between genders, impacting traits such as rust resistance [20], stolon growth ability [21], nitrogen form preference [19] and transcriptomics and metabolomics [22]. Research on gender differences in *B. dactyloides* regarding drought resistance has largely focused on physiological aspects, with limited exploration of its molecular regulatory mechanisms under drought stress. This study utilizes SMRT sequencing to obtain the complete transcriptome sequence of *B. dactyloides*, serving as a reference for subsequent NGS sequencing. The research aims to investigate the molecular regulatory mechanisms governing *B. dactyloides*’ drought resistance and recovery under drought stress, specifically elucidating gender differences. This study provides a theoretical foundation for understanding *B. dactyloides*’ drought resistance and investigating the response mechanisms of dioecious plants to abiotic stress.

## 2. Results

### 2.1. Analysis of B. dactyloides Physiological States and Phenotypic Changes

**Figure 1:**
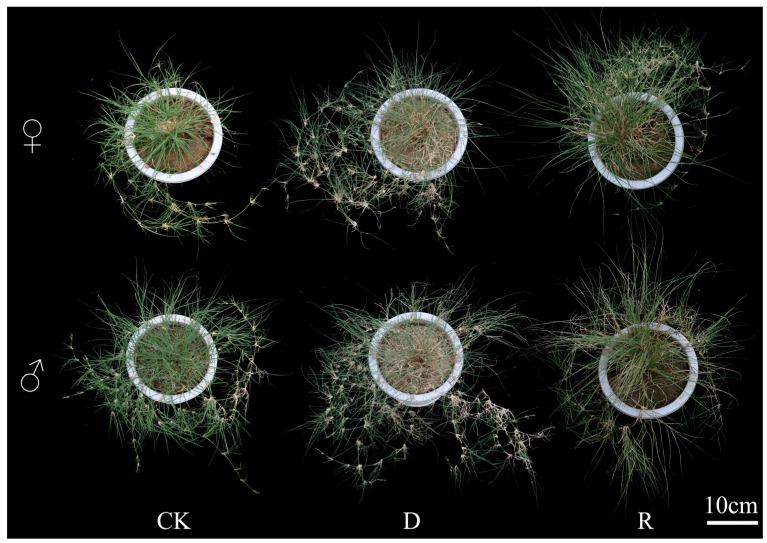
The phenotypic changes in *B. dactyloides*. CK—normal growth, D—drought treatment for 14 days, R—rewatering treatment for seven days, ♀—female *B. dactyloides*, and ♂—male *B. dactyloides*, the same below.

The wilting of *B. dactyloides* occurred obviously after drought and recovered after rehydration. The wilting degree of male *B. dactyloides* was greater than female *B. dactyloides* (Figure 1).

**Figure 2:**
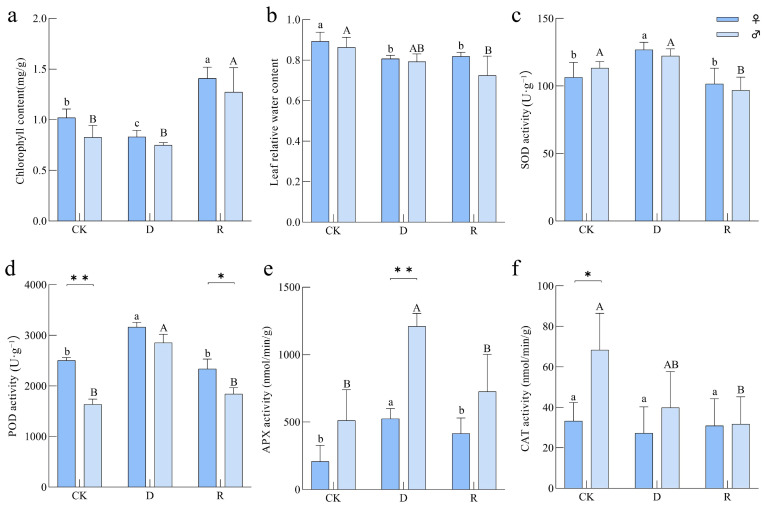
Changes in chlorophyll and relative water content of *B. dactyloides* leaves. (**a**) Changes in chlorophyll; (**b**) leaf relative water content change; (**c**) SOD activity; (**d**) POD activity; (**e**) APX activity; (**f**) CAT activity. Lowercase letters indicate differences between different treatments of female *B. dactyloides* (*p* < 0.05). Capital letters indicate differences between different treatments of male *B. dactyloides* (*p* < 0.05). * indicate differences between female and male *B. dactyloides* (*p* < 0.05). ** indicate differences between female and male *B. dactyloides* (*p* < 0.01).

Chlorophyll is a crucial indicator reflecting plant photosynthesis and growth status. In this study, the trends in chlorophyll content changes were similar in both male and female *B. dactyloides* plants across the three treatments. Chlorophyll content was lowest under drought treatment, significantly increasing after rewatering, and notably higher than under the control treatment (CK) (Figure 2a). The relative water content (RWC) of female *B. dactyloides* leaves significantly decreased under drought treatment but showed a slight increase after rewatering. In contrast, the decrease in RWC of male *B. dactyloides* plants was not significant after drought and continued to decrease after rewatering (Figure 2b). It suggests that male *B. dactyloides* plants may have lower water retention capacity compared to females. The activities of POD, SOD, and APX enzymes in both male and female *B. dactyloides* plants peaked under drought conditions (Figure 2c–e). Specifically, the POD activity of female plants was significantly higher than that of males after CK and rewatering treatments, with no significant change in CAT activity (Figure 2f). Under drought stress, APX activity in male *B. dactyloides* plants was significantly higher than in females, while CAT activity was notably higher under CK conditions compared to the other two treatments (Figure 2f).

### 2.2. GO and KEGG Pathway Analysis of DEGs between Female and Male B. dactyloides

NGS was performed with reference to previous laboratory SMRT results [22]. Transcriptome sequencing of 18 samples was completed with a Q30 score of 85%, enabling subsequent analysis of differentially expressed genes (DEGs), their number, and functional annotation (Table S1). Through the correlation heat map clustering analysis of transcript expression (Figure S1a), it is obvious that the same gender and the same treatment are clustered together, demonstrating the reliability of biological replication in this study. TBtools was used to classify all the differential genes by Ven diagram (Figure S1b,c). The Ven diagram showed that compared with male plants, female plants had more DEGs involved in the reaction during drought stress and rewatering, while male plants had more DEGs activated and expressed after rewatering.

**Figure 3:**
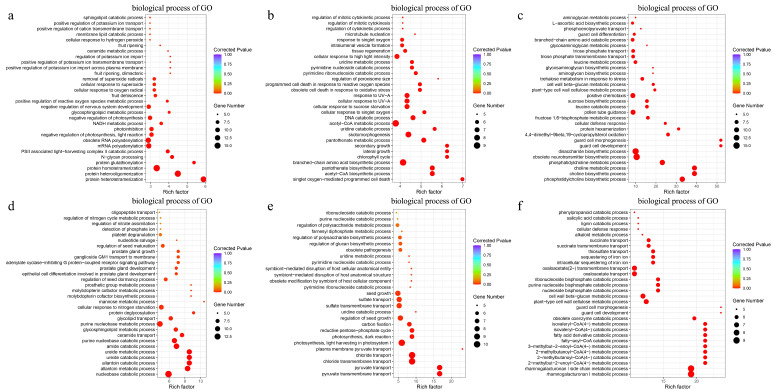
GO enrichment of DEGs in drought and rewater treatment between female and male *B. dactyloides* in biological process. (**a**) GO enrichment of DEGs unique to female CK vs. D; (**b**) GO enrichment of DEGs unique to female D vs. R; (**c**) GO enrichment of DEGs among to female CK vs. D vs. R; (**d**) GO enrichment of DEGs unique to male CK vs. D; (**e**) GO enrichment of DEGs unique to male D vs. R; (**f**) GO enrichment of DEGs among to male CK vs. D vs. R.

From the results of the analysis results, it can be seen that the 3688 DEGs unique to CK vs. D female plants are more enriched in PSII-associated light-harvesting complex II catalytic processes, N-glycan processing, protein glutathioneization, and protein heterotrimerization (Figure 3a). The 2757 DEGs unique to CK vs. D male plants are more enriched in protein metabolism, ureide metabolic process, and allantoin catalytic process (Figure 3d), while the 5000 DEGs unique to D vs. R female plants are more enriched in singlet oxygen-mediated programmed cell death, secondary growth, lateral growth, chlorophyll cycle (Figure 3b). The 2245 DEGs unique to D vs. R male plants are more enriched in plasma membrane pyruvate transport, pyruvate transport, and glucose transport (Figure 3e). The 2207 DEGs unique to female plants are more enriched in guard cell morphogenesis, guard cell development, and fatty acid derivative catalytic process (Figure 3c). The 1134 DEGs unique to male plants are more enriched in guard cell morphogenesis, guard cell development, and cellular defense response (Figure 3f). It indicates that there are significant differences in GO enrichment between female and male *B. dactyloides*.

**Figure 4:**
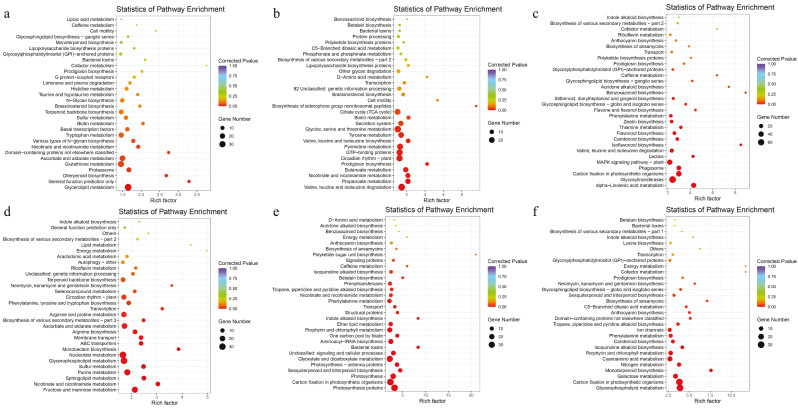
KEGG pathway enrichment of DEGs unique to *B. dactyloides*. (**a**) KEGG pathway enrichment of DEGs unique to female CK vs. D; (**b**) KEGG pathway enrichment of DEGs unique to female D vs. R; (**c**) KEGG pathway enrichment of DEGs among to female CK vs. D, CK vs. R, D vs. R; (**d**) KEGG pathway enrichment of DEGs unique to male CK vs. D. (**e**) KEGG pathway enrichment of DEGs unique to male D vs. R; (**f**) KEGG pathway enrichment of DEGs among to male CK vs. D, CK vs. R, D vs. R.

KEGG analysis of unique DEGs in female CK vs. D revealed enrichment in glycerolipid metabolism and general function prediction only (Figure 4a). Meanwhile, the 2757 DEGs unique to male plants in the same comparison were enriched in pathways such as nicotinate and nicotinamide metabolism, and transcription (Figure 4d). In the comparison D vs. R, the 5000 DEGs unique to female plants were enriched in pathways including prodigiosin biosynthesis, valine, leucine, and isoleucine biosynthesis, propanoate metabolism, and butanoate metabolism (Figure 4b). On the other hand, the 2245 DEGs unique to male plants were enriched in pathways such as indole alkaloid biosynthesis, sesquiterpenoid and triterpenoid biosynthesis, and photosynthesis proteins (Figure 4e). For the comparison CK vs. D vs. R, KEGG analysis showed that the 2207 DEGs unique to female plants were enriched in pathways such as isoflavonoid biosynthesis, alpha-linolenic acid metabolism, and MAPK signaling pathway-plant (Figure 4c). Conversely, the 1134 DEGs unique to male plants in the same comparison were enriched in pathways including monoterpenoid biosynthesis, carbon fixation in photosynthetic organisms, and glycerophospholipid metabolism (Figure 4f). These results underscore the differential enrichment of metabolic pathways in female and male *B. dactyloides* plants under drought stress and rewatering conditions, highlighting potential gender-specific responses at the molecular level.

### 2.3. Difference of Plant Hormone Signal Transduction and Biosynthesis Pathway between Female and Male B. dactyloides

**Figure 5:**
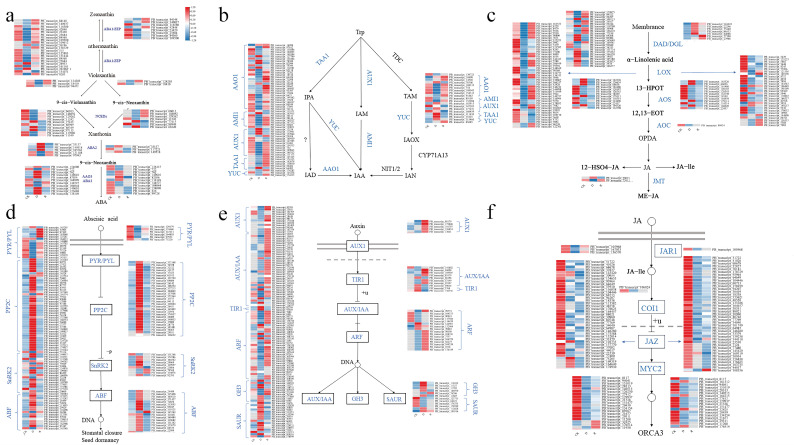
Analysis of DEGs related to plant hormone biosynthesis and signal transduction pathways. (**a**) Expression patterns of DEGs involved in ABA biosynthesis pathways (**b**) Expression patterns of DEGs involved in ABA signaling pathways; (**c**) Expression patterns of DEGs involved in auxin biosynthesis pathways; (**d**) Expression patterns of DEGs involved in auxin signaling pathways; (**e**) Expression patterns of DEGs involved in JA biosynthesis pathways; (**f**) Expression patterns of DEGs involved in JA signaling pathways. The left side of the pathway is the female plant and the right side is the male plant.

We conducted specific analyses to clarify the differences in hormone signaling mechanisms between male and female *B. dactyloides* under drought stress and after rewatering. In the ABA biosynthesis pathway (Figure 5a), female *B. dactyloides* exhibited activation and expression of numerous genes in zeaxanthin epoxidase (ZEP). During drought stress, most genes were downregulated, with only four DEGs significantly upregulated. Genes encoding 9-cisepoxycarotenoid dioxygenase (NCEDs) and xanthoxin dehydrogenase (ABA2) were induced, promoting ABA biosynthesis. Specifically, NCEDs showed upregulation in a few genes, while ABA2, abscisic-aldehyde oxidase (AAO3), and alpha-ionylideneethane synthase aba3 (ABA3) had more upregulated than downregulated DEGs. After rewatering, the gene expression pattern reversed compared to drought stress. Similarly, male *B. dactyloides* showed a pattern in the ABA biosynthesis pathway akin to females (Figure 5d), with downregulation in ZEP and upregulation in ABA2, AAO3, and ABA3. However, the number of DEGs involved in regulating this pathway was fewer in males compared to females, suggesting a slower ABA signal transduction rate and potentially weaker drought response in males. Regarding IAA, a crucial hormone for plant growth, drought stress inhibited its biosynthesis, which recovered after rewatering (Figure 5b). In females, AMI and YUC genes exhibited downregulated expression under drought stress. Conversely, in males, the indole-3-pyruvate (IPA) pathway showed upregulated gene expression during drought, though overall lower compared to females. This difference may contribute to varying drought resistance between male and female plants. In the IAA signaling pathway (Figure 5e), female *B. dactyloides* displayed significant activation and expression of genes under drought stress compared to CK conditions. Genes in auxin influx transporter (AUX) were downregulated, while those in transport inhibitor response protein 1 (TIR1) were upregulated. Genes in AUX/IAA were largely downregulated, while those in auxin response factor (ARF), Gretchen Hagen 3 (GH3), and small auxin up RNA (SAUR) were predominantly upregulated. After rewatering, gene expression patterns reversed, indicating recovery in female plants. Male plants showed a similar pattern in the IAA signaling pathway but with lower gene expression levels compared to females, reinforcing their potentially weaker drought resistance. In the jasmonic acid (JA) biosynthesis pathway (Figure 5c), both male and female *B. dactyloides* exhibited differential gene regulation under drought stress. Females showed upregulated gene expression in jasmonic acid carboxyl methyltransferase (JMT), while males showed upregulation in allene oxide cyclase (AOC). After rewatering, females displayed significant upregulation of lipoxygenase (LOX) gene expression, whereas males showed downregulation, suggesting faster JA synthesis recovery in females. In the JA signaling pathway (Figure 5f), both genders exhibited similar trends, with drought significantly inhibiting gene expression of the core inhibitory factor jasmonate ZIM domain (JAZ), which remained downregulated after rewatering. This pattern accelerates JA signal transduction to cope with drought stress. These findings underscore the complex and gender-specific responses of *B. dactyloides* plants to drought stress at the molecular level, potentially influencing their overall resilience and recovery capabilities.

### 2.4. DEGs Involved in Photosynthetic Pathways between Female and Male B. dactyloides

Analyzing the DEGs involved in photosynthesis, we found notable differences between female and male plants of *B. dactyloides* in response to drought stress and rewatering. In female plants (Figure S2), after rewatering, genes encoding the Light-harvesting chlorophyll a/b binding (LHC) Lhca1-5 and Lhcb1-7 were significantly upregulated compared to drought conditions. Specifically, Lhca1 showed some downregulated genes under drought stress but predominantly upregulated genes after rewatering. Conversely, compared to CK, drought stress inhibited Lhca1-5 and Lhcb1-7, with only Lhca1 showing upregulated genes. Rewatering resulted in a mixed response with both upregulated and downregulated genes, particularly in Lhca2, Lhcb1, Lhcb3, and Lhcb5. In male plants (Figure S2), gene expression was generally inhibited under drought stress compared to CK, particularly in Lhca1, Lhca2, Lhca4, and Lhca5, with no gene expression detected in other proteins. Rewatering treatment compared to CK showed upregulated genes in all proteins except Lhca3. Interestingly, compared to drought stress, rewatering treatment upregulated gene expression in all proteins except Lhcb2. These results indicate that female *B. dactyloides* plants experience more pronounced inhibition of photosynthesis under drought stress compared to males. The differential responses in gene expression related to LHC proteins highlight potential gender-specific strategies in coping with water scarcity and recovery after rewatering.

### 2.5. WGCNA Analysis Identified Key Drought Response Genes in Female and Male B. dactyloides

**Figure 6:**
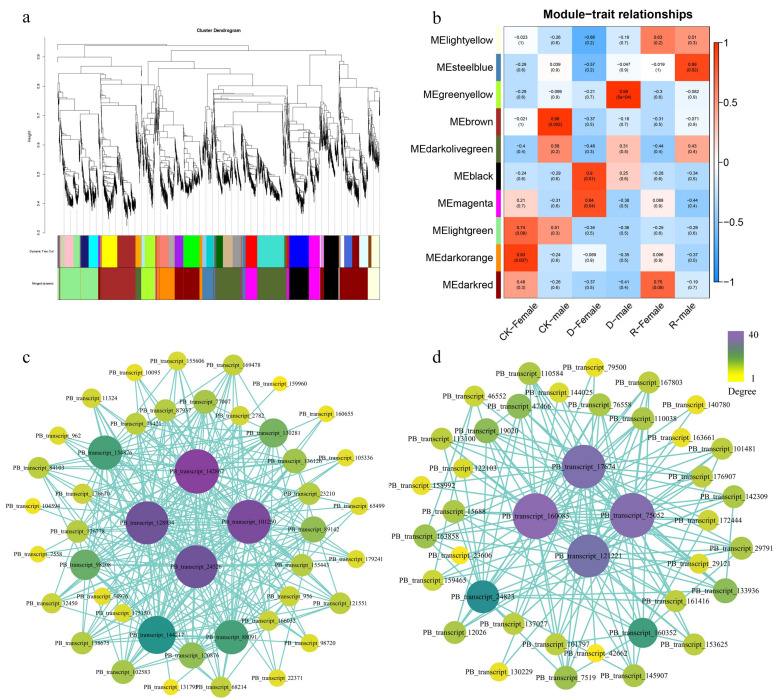
Weighted gene co-expression network establishment and correlation analysis (**a**) Gene clustering tree (dendrogram); (**b**) The heat map shows the correlation between the identified modules and different periods; (**c**) A network analysis of the hub genes in magenta modules; (**d**) A network analysis of the hub genes in greenyellow modules.

We conducted WGCNA analysis using biomarker Cloud to understand the potential molecular regulatory mechanisms between male and female plants of *B. dactyloides* under drought stress. We selected all DEGs (FPKM > 1) from 18 samples and used different treatments as reference traits. The minimum gene module was 30, with a module similarity of 0.25. The cluster tree diagram identified 10 different modules (Figure 6a), a correlation heatmap was created between modules and traits, with the greenyellow module showing a positive correlation with drought treatment in D-male and the magenta module showing a positive correlation with drought treatment in D-female. The steelblue module shows a positive correlation with rewatering treatment in R-male. The darkorange module and brown module showing a positive correlation with CK treatment in CK-female (Figure 6b). Considering the correlation between modules and traits and the proportion of genes within the modules, we chose the magenta module and greenyellow module as key modules for the response to drought stress and drought recovery ability of *B. dactyloides*. Using Cytoscape screen out eight key genes for two modules: *PB_transcript_160085*, *PB_transcript_75052*, *PB_transcript_121221*, *PB_transcript_17674*, *PB_transcript_142867*, *PB_transcript_128934*, *PB_transcript_24526*, *PB_transcript_101250*. (Figure 6c,d).

### 2.6. Yeast Verification of Key Genes under Drought Stress

**Figure 7:**
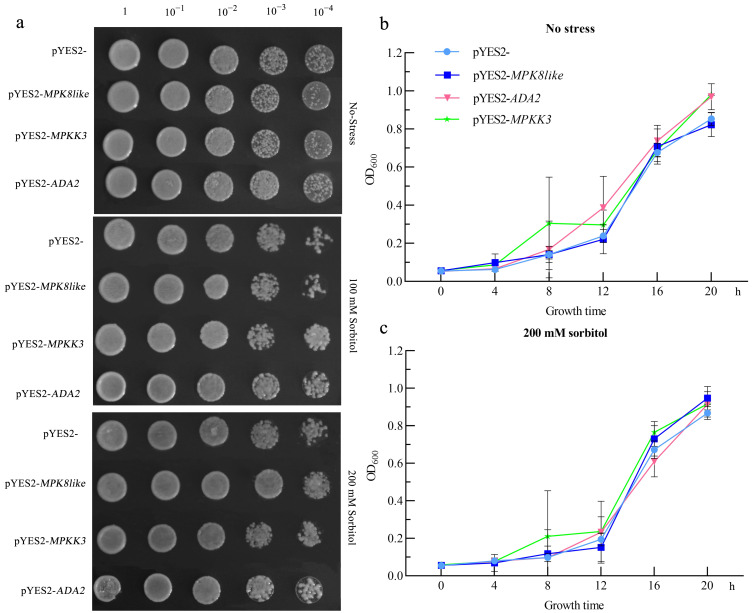
Changes in growth of transgenic yeast under different drought conditions (**a**) The change in yeast growth state under different conditions; (**b**) Change in yeast growth in time without stress; (**c**) Change in yeast growth in time under 200 mM sorbitol.

The FPKM of eight key genes were calculated by transcriptome data. The results showed that the expression levels of eight genes were different under different treatments, and there were significant differences between male and female strains (Figure S3). Based on their transcription factor effects, gene function annotation information (Table 1), and their FPKM in different treatments, we further screened out three key genes: *PB_transcript_75052*, *PB_transcript_128934*, *PB_transcript_101250*. Sequence comparison from the NCBI gene database showed that three genes named *MPK8-like*, *MPKK3*, and *ADA2*. The functional verification of *BdMPK8-like*, *BdMPKK3*, and *BdADA2* show that the growth state of pYES2- was significantly stronger than that of pYES2-*MPK8-like* and pYES2-*BdMPKK3* in the medium without sorbitol (Figure 7a). The growth state of pYES2-*BdADA2* was similar to pYES2- with no significant change. Under moderate drought stress, the growth state of pYES2-*BdMPK8* remained weaker than that of pYES2-, while the growth states of pYES2-*BdMPKK3* and pYES2-*BdADA2* were stronger than that of pYES2- (Figure 7a). Under severe drought stress, it was observed that the growth state of pYES2-*BdMPK8-like* was stronger than that of pYES2-, while the growth states of the other two yeasts were similar to those of pYES2- (Figure 7a). Four types of transgenic yeast were cultured in a liquid medium, and their growth curves were determined. From the growth curves, it can be seen that the growth of pYES2- *MPK8-like* was lower than pYES2- in a normal environment (Figure 7b), while the growth was significantly higher than pYES2- in a severe stress environment (Figure 7c). Based on the growth phenotype and growth curves of transgenic yeast, *BdMPK8-like* may be a drought resistance-related gene in *B. dactyloides*, which helps improve its drought resistance.

## 3. Discussion

*B. dactyloides*, a warm-season lawn grass known for its robust stress resistance, low fertilizer requirements, and rapid turfgrass establishment [23], was subjected to simulated drought conditions in this study to investigate its internal regulatory mechanisms through transcriptional analysis. Female *B. dactyloides* showed stronger growth and recovery during drought compared to males (Figure 1). This gender disparity in response is a common trait among dioecious plants [24]. Both male and female plants exhibited decreased chlorophyll content and RWC under drought stress, with recovery upon rewatering (Figure 2), consistent with findings by Patanè [25]. However, distinct variations were observed between genders, highlighting stronger drought resistance and recovery abilities in females based on phenotypic and physiological analyses. To investigate the molecular regulatory mechanisms of *B. dactyloides* under drought stress in male and female plants, we employed SMRT and NGS analyses. The repeatability analysis of the samples validated the scientific rigor of our experimental design [26]. Cluster analysis of 18 samples revealed that samples treated identically clustered together, with significant differentiation observed between genders (Figure S1). Additionally, qRT-PCR results showed that the relative gene expression of 10 genes has the same trend as that of RNA-seq (Figure S4). It indicates that RNA sequencing data could be effectively utilized for subsequent bioinformatics analyses. Drought stress triggers extensive changes in gene expression, reflecting diverse regulatory mechanisms in plants [27]. GO analysis highlighted distinct responses in DEGs between male and female *B. dactyloides* under drought stress. Female plants exhibited heightened involvement in processes such as photosynthesis and energy metabolism, whereas males were more engaged in stress response regulation (Figure 3). Notably, other studies have indicated that allantoin plays a pivotal role in nitrogen transport, storage, and reuse in plants [28]. Under drought conditions, plants accumulated higher levels of allantoin, triggering robust stress responses [29]. Following rewatering, DEGs in female plants predominantly focused on plant regeneration post-drought stress, emphasizing biomass accumulation and photosynthesis. In contrast, male plants showed enrichment of DEGs related to carbon metabolism, particularly in the transport and accumulation of pyruvate critical substrate in sugar degradation processes [30].

Drought can cause changes in protective cells to regulate stomatal size in response to drought stress [31]. Analysis across three treatments revealed common DEGs involved in regulating morphological changes, growth, and development of guard cells in both male and female plants. KEGG analysis revealed that under drought stress, female *B. dactyloides* showed an enrichment of DEGs in glycerolipid metabolism. This pathway primarily contributes to energy metabolism, signal transduction, and stress response [32]. In contrast, male plants showed enriched DEGs in nicotinate, and nicotinamide metabolism and transcription pathways. Nicotinate and nicotinamide metabolism are vital for auxin production and interact with ABA signaling pathways regulating plant stress resistance [33]. After rewatering, DEGs in female *B. dactyloides* predominantly enriched pathways such as propanoate and butanoate metabolism, essential for plant growth and stress resistance [34]. Meanwhile, male plants exhibited enrichment in pathways related to auxin synthesis and photosynthesis (Figure 4). These differential enrichments suggest distinct adaptive strategies between genders. Further KEGG and GO analyses indicated that female *B. dactyloides* were more enriched in the related processes mediated by flavonoids. Flavonoids are mainly involved in antioxidant activity [35], cell membrane fluidity [36], and signal transduction [37]. The male plants were more enriched in transcription and pathways associated with hormonal activity. These divergent gene expression patterns between genders underlie the variability in drought resistance observed in *B. dactyloides*.

Plant hormones play pivotal roles in stress responses [38]. The ABA signaling pathway involves PYR/PYL receptors, PP2C phosphatases, and SnRK2 kinases [39,40]. Under stress, ABA interacts with PP2C. This interaction leads to the dissociation of PP2C-SnRK complexes, activating SnRK2 kinases. Activated SnRK2 kinases phosphorylate various substrates, including ABRE-binding proteins (ABRE-b), thereby regulating plant stress responses and adaptation [41]. In this study, drought stress induced significant expression of PP2C and SnRK2 genes in *B. dactyloides*, similar to findings in other plants like *Carex moorcroftii* [10], *Malus pumila* [42], and *Triticum aestivum* [43]. ABA biosynthesis primarily occurs in plant leaves and root caps [44]. ABA plays critical roles in drought response, such as regulating stomatal closure [45], managing reactive oxygen species [46], and influencing leaf senescence [47]. Under drought stress conditions, numerous genes in the ABA synthesis pathway are activated. Specifically, AAO3 catalyzes the rapid conversion of abscisic aldehyde to ABA, enhancing drought resistance in plants [48]. In this study, upregulation of AAO3 genes was observed in *B. dactyloides*. Female *B. dactyloides* plants exhibited more genes involved in ABA signal transduction and synthesis pathways compared to males, suggesting a potentially more intricate molecular regulatory mechanism in response to drought stress (Figure 5a,d). Auxin signaling involves three main protein receptors: TIR1/AFB, transcriptional repressors AUX/IAA, and auxin response factors ARF [49]. In this study, drought stress significantly induced gene expression in the IAA signaling pathway of *B. dactyloides*. Under drought conditions, genes such as AUX1, AUX/IAA, and ARF were predominantly downregulated, while TIR1 expression was upregulated. TIR1, an auxin receptor protein, promotes AUX/IAA degradation, thereby enhancing auxin signaling [50]. Liberation of ARF promotes the upregulation of early auxin response genes like *GH3* and *SAUR*. Studies have demonstrated that drought stress upregulates *GH3* and *SAUR* expression [51]. Overall, when *B. dactyloides* was under drought stress, a large number of genes in the IAA signaling pathway were down-regulated, with opposite gene expression patterns observed after rewatering. Similar results were found in *Eriobotrya japonica* [52]. Auxin biosynthesis occurs through pathways including IPA, indole-3-acetamide (IAM), and indole-3-acetaldoxime (IAOx) [53]. While the IAOx pathway is less common and found in specific plant families like Brassicaceae, Poaceae, and Musaceae, the IPA pathway is fundamental in plant auxin synthesis [54]. Drought inhibits auxin synthesis pathway gene expression, but notably, genes in the IPA pathway of auxin synthesis are upregulated in male *B. dactyloides* plants under drought conditions. This suggests that drought promotes auxin synthesis specifically in male plants, highlighting gender-specific responses in *B. dactyloides* to environmental stresses (Figure 5b,e). JA-Ile promotes the expression of coronatine-insensitive 1 (COI1), which mediates the degradation of JAZ proteins, thereby activating JA signal transduction [45]. In this study, most genes in the JA signaling pathway exhibited upregulation during control conditions but showed downregulation after drought stress. Similar findings were reported by Chen et al. in *Poa pratensis* [55], where early drought stress downregulated JA signaling genes, suggesting a role in adapting to prolonged drought stress. Yao et al. also observed the upregulation of aboveground JA-related genes during dry seasons. Interestingly, COI1 activation was observed only in female plants, indicating gender-specific responses in *B. dactyloides* [56]. Moreover, female plants showed a distinct pattern of JAZ gene expression compared to males, with more DEGs involved and a higher number of upregulated genes. The main pathway for JA synthesis is the octadecanoid pathway, initiated by α-linolenic acid released from chloroplasts [57]. Unsaturated fatty acids are converted into 12-oxo-phytodienoic acid (12-OPDA), which undergoes further oxidation to form JA. JA-Ile is the biologically active form of JA [58]. In this study, while *B. dactyloides* under drought stress, genes encoding enzymes like DAD/DGL involved in α-linolenic acid metabolism are significantly upregulated, boosting its production in chloroplasts, but key enzymes in JA synthesis, such as *AOS, AOC*, and *OPR3*, show downregulation, indicating that drought stress inhibits JA biosynthesis (Figure 5c,f). This phenomenon is possibly influenced by crosstalk with ABA. Wang et al. suggested that JA enhances wheat drought resistance upstream of ABA [59], while Peian et al. demonstrated that exogenous ABA application inhibits endogenous JA levels in *Fragaria × ananassa* Duch. [60].

The accumulation of reactive oxygen species (ROS) and reduced CO_2_ influx can inhibit photosynthesis rates under drought stress [61]. LHC proteins are pigment proteins crucial for capturing light energy and facilitating photochemical reactions in plants. They play vital roles in light energy absorption, transmission, maintaining membrane integrity, and adapting to environmental changes [62]. Under drought stress, both male and female *B. dactyloides* plants exhibit significant downregulation of LHC protein genes. However, female plants of *B. dactyloides* differ from males in that they show more pronounced downregulation of LHC proteins. This suggests that female plants allocate more genes to regulate photosynthesis during drought stress (Figure S2), thereby enhancing their drought resistance. Similar observations were noted in buckwheat (*Fagopyrum esculentum*), where the downregulation of LHC protein genes was reported under drought conditions [63]. After rewatering, genes encoding LHC proteins in both male and female *B. dactyloides* plants show significant upregulation. This upregulation reflects the plants’ increased need for photosynthesis to recover from drought-induced damage. Interestingly, there were no significant differences in the gene expression patterns of LHC proteins between male and female *B. dactyloides* plants after rewatering.

**Figure 8:**
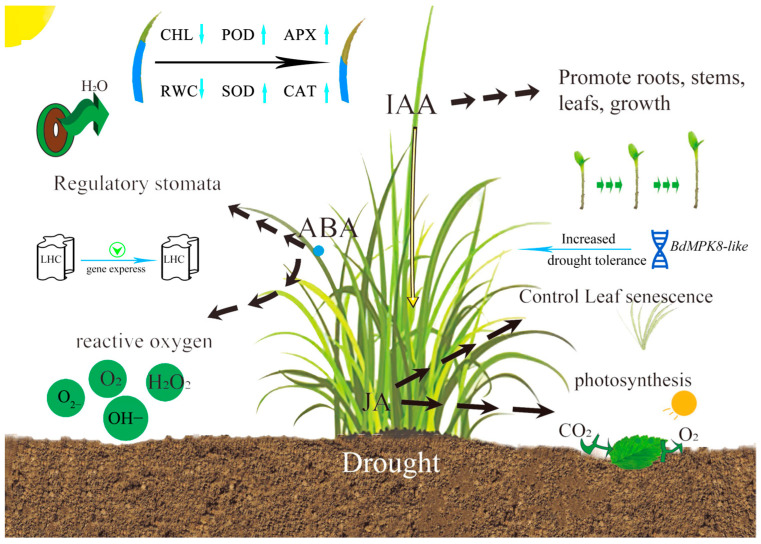
The model of *B. dactyloides* under drought condition.

The use of transcriptomics technology to mine plant resistance genes has become a hot spot [64]. In this study, we applied WGCNA to analyze transcription factor-related genes in *B. dactyloides* and identified key genes (Figure 6). It was confirmed that the *BdMPK8-like* gene could enhance drought resistance of *B. dactyloides* by yeast transformation experiment (Figure 7). The MAPK cascades play pivotal roles in plant stress responses [65]. Mitogen-activated protein kinases (MAPKs or MPKs) diversify plant signaling pathways, enabling plants to respond to external signals through phosphorylation processes crucial for growth and development in complex environments [66]. MAPKs are known for their essential roles in plant development and stress responses [65,67]. They are associated with various kinases that enrich plant signal transduction pathways, adapting plants to complex environmental conditions [68]. *MAPK* family members have been extensively studied in *Arabidopsis* [69], *Oryza sativa* [70], and *Fragaria vesca* [71]. For instance, *AtMPK6* and *AtMPK4* in *Arabidopsis thaliana* mediate tolerance to low temperatures and salt stress [72]. The involvement of MAPKs in plant responses to drought stress has attracted considerable attention. In *Arachis hypogaea*, the *AhMAPK13* gene is significantly induced under drought stress [73]. In *Gossypium hirsutum*, the *GhMAP3K14*-*GhMKK11*-*GhMPK31* MAPK cascade responds to drought stress [74]. *BdMPK8-like* identified in *B. dactyloides* has been validated as a critical gene for drought resistance. Similarly, in rice, *MAPK10.2* acts at the intersection of two MAPK cascades, enhancing drought resistance and drought tolerance [75]. Additionally, transgenic plants overexpressing *StMAPK11* exhibit increased activities of antioxidant enzymes (SOD, CAT, POD, and PRO) under drought stress, significantly improving drought resistance [76]. As shown in Figure 8, based on our research results, we established a model figure of the changes in antioxidant enzymes and hormones in *B. dactyloides* under drought stress.

## 4. Materials and Methods

### 4.1. Test Materials

*B. dactyloides* (cv. ‘Texoka’) were sown in the greenhouse at the Institute of Grassland, Flower and Landscape Ecology, Beijing Academy of Agriculture and Forestry (Beijing, China) in August 2020. By June 2021, the plants had bloomed and based on the discernible differences in flower types between female and male plants post-flowering, sex distinctions were identified. Subsequently, female and male plants were selectively propagated and expanded to establish a single-sex clone population with uniform genetic background and genotype by February 2022.

### 4.2. Experimental Design

In June 2023, a flower pot measuring 19 cm in top diameter, 10 cm in bottom diameter, and 15 cm in height was used for planting. Each pot contained 6–7 tillers of either female (♀) or male (♂) *B. dactyloides* asexual seedlings as individual plants. Garden soil was used as the planting medium. This study comprised two gender groups: female (♀) and male (♂), each subjected to three treatments: adequate watering (watered until seepage from the bottom; designated as ♀ CK and ♂ CK), natural drought treatment for 14 days (no watering for 14 days; ♀ D and ♂ D), and rewatering treatment for 7 days after drought (watered until seepage from the bottom; ♀ R and ♂ R). Each treatment was replicated three times, resulting in a total of 18 samples. Leaf samples were collected on the first day of drought, the 14th day of drought, and the 7th day of rewatering. Fresh leaf samples were immediately used for measuring relative water content and chlorophyll content, while the remaining samples were stored in a −80 °C freezer for subsequent analyses.

### 4.3. Determination of Chlorophyll Content

After weighing about 0.05 g of leaves, the specific weight was recorded, and the leaves were put into a 10 mL centrifuge tube and added with 8 mL 95% ethanol. Then store in darkness for 24 h. The content of chlorophyll (Chl) is calculated using an enzyme-linked immunosorbent assay (Multiskan FC, Thermo, Waltham MA, USA) at 665 nm, 649 nm, and 470 nm.

### 4.4. Determination of Relative Leaf Water Content

The sample was weighed at 0.1 g and its fresh weight *WF* was recorded. Put the designated blade into a 50 mL triangular bottle, fill it with distilled water, seal it, and leave it for 24 h. Remove the leaves quickly and gently dry the surface water, measure the saturated weight *WT*. Put the saturated leaves into the aluminum box and dry them at 105 °C for 15 min. Bake at 80 °C to constant weight (at least 72 h). After removal, cool naturally and weigh dry *WD*.

(1) $$\mathrm{relative}\;\mathrm{leaf}\;\mathrm{water}\;\mathrm{content},\;\mathrm{RWC}\;\left(\%\right)=\frac{WF-WD}{WT-WD}\times 100\%$$

### 4.5. Antioxidant Enzyme Assay

The activities of SOD, POD, CAT, and APX were measured using reagent kits (Suzhou Keming Biotechnology company, Suzhou, China) according to the manufacturer’s instructions. Three biological repetitions were adopted for each of the above physiological measurements. The absorbance values were measured and enzyme activity was calculated using an enzyme-linked immunosorbent assay (Multiskan FC, Thermo, Waltham, MA, USA) and a UV spectrophotometer (UV2600, Techcomp, Hong Kong, China) at 560 nm, 470 nm, 240 nm, and 290 nm.

### 4.6. Illumina cDNA Library Construction and Sequencing

We sampled the female and male *B. dactyloides* with drought 0 days as CK, drought 14 days, and rehydration 7 days as treatment, respectively. Three biological replicates were performed for each treatment. A total of 18 RNA seq libraries were constructed for Illumina sequencing and the prepared RNA samples were evaluated. NGS was conducted at Illumina Novaseq 6000 (San Diego, CA, USA). Then, we cut the sequencing adapter and primer sequence in the reads and filtered low-quality isomer (LQ) data. Obtained high-quality reads (clean data) to calculate Q30, GC content, and sequence repeat levels.

### 4.7. DEGs Expression Analysis and Gene Annotation

BLAST (http://blast.ncbi.nlm.nih.gov/Blast.cgi accessed on 1 September 2023) software (version 2.2.26) was used to compare the obtained nonredundant transcripts with KEGG databases to obtain annotated information of the transcripts [77]. The fragments per kilobase of transcript per million fragments (FPKM) method was used to detect whether the number of mapped reads and the length of transcripts in the sample had reached the gene expression level [78]. DEseq2 was used for screening DEGs [79]. In this study, the differences among the 6 group samples (female CK vs. female D, female CK vs. female R, female D vs. female R, male CK vs. male D, male CK vs. male R, male D vs. male R) were calculated. The KEGG and GO analysis of DEGs specific to different groups was performed by using TBtools (version 1.33225.0.0) software. And R language was employed for generating visual representations of data.

### 4.8. cDNA Synthesis and Quantitative Reverse Transcription (qRT-PCR)

The cDNA reverse transcription kit (TaKaRa, Dalian, China) was used to generate cDNA. Primer design was facilitated using Primer Premier 5 software. For quantitative validation, qRT-PCR was performed on the Bio-rad CFX Connect 96-well fluorescence quantitative PCR platform (Bio-Rad, Hercules, CA, USA), utilizing SYBR Green PCR Master mix (TaKaRa, Dalian, China). Actin was used as the reference gene, and the expression was calculated with the 2^−ΔΔCT^ method.

### 4.9. Gene Expression Quantification

Gene expression level could be quantified by Kallisto [80] based on transcripts generated from CCS data. To eliminate the affected gene length and data size on gene expression level, FPKM (Fragments Per Kilobase of transcript per Million mapped reads) is applied here as a standard method to estimate gene expression level. The formula for calculating FPKM is shown as below:

(2) $$FPKM=\frac{cDNA\;Fragments}{Mapped\;Fragments\;(Millions)*TranscriptLength(kb)}$$

In the formula, *cDNA Fragments* stands for the number of reads (PE reads) mapped to the specific transcript; *Mapped Fragments (Millions)* stands for the number of total mapped reads (in 10^6^). *Transcript Length* (kb): the length of transcripts (in 10^3^ bp).

### 4.10. Gene Expression Pattern Analysis

WGCNA analysis was performed using BMK Cloud, Genes with FPKM > 1 were used as the original data, and genes with similar expression patterns were clustered based on the correlation coefficient of gene expression, thereby constructing gene modules and Cytoscape_v3.9.1 was used for key gene screening. The gene expression heat map was generated using TBtools (version 1.33225.0.0) software, with genes that have three replicates with FPKM > 2 in at least one stage, and with an FDR < 0.05 between any two adjacent stages being plotted on the map.

### 4.11. Verification of Gene Function

The drought resistance genes *BdMPK8-like*, *BdMPKK3*, and *BdADA2* of *B. dactyloides* were selected through WGCNA and Cytoscape. The target plasmid was amplified by PCR, purified, and then single-cut with *EcoR* I enzyme to connect with the digested pYES2 vector. The connection product was transformed into DH5α and selected on Amp-containing LB plates. Positive colonies were verified by PCR and sequenced to confirm correct plasmids (pYES2-*BdMPK8-like*, pYES2-*BdMPKK3*, pYES2-*BdADA2*).

For yeast transformation, Y187 receptive cells were prepared and transformed with the plasmids. The transformants were plated on SD/-Ura plates and cultured to select positive clones. Single colonies of positive recombinant plasmid and control plasmid pYES2 were selected and expanded in SD/-Ura liquid screening medium to achieve OD_600_ of 0.5. After the bacterial solution was diluted to 10, 10^−1^, 10^−2^, 10^−3^, 10^−4^ times, the coating plate was cultured on SD/-Ura, SD/-Ura with 100 mM sorbitol, and SD/-Ura with 200 mM sorbitol, and the yeast growth phenotype was observed at 30 °C for 3d.

## 5. Conclusions

This study analyzed the transcriptome changes in male and female *B. dactyloides* plants under drought stress and rewatering treatment and found differences in molecular regulatory mechanisms between female and male *B. dactyloides* plants in response to drought stress. Our research results indicated that the growth status of female *B. dactyloides* plants is stronger than that of male plants, and the drought resistance and drought recovery ability of female plants are stronger than male plants. From a molecular regulatory perspective, there is a significant difference in the number of differentially expressed genes between male and female *B. dactyloides* plants under drought stress and rewatering conditions. And we found drought resistance genes such as *BdMPK8-like* in *B. dactyloides*. This study provided a theoretical basis for the subsequent practical application of *B. dactyloides* and related breeding work and enriched the research on the stress resistance of dioecious plants.

## Figures and Tables

**Table 1:** The function annotation of key genes.

Transcript ID	TF	Eggnog_Class_Annotation	NR_Annotation
*PB_transcript_160085*	TCP	--	transcription factor TCP7
*PB_transcript_75052*	MAPK	Signal transduction mechanisms	hypothetical protein
*PB_transcript_121221*	MAPK	Signal transduction mechanisms	hypothetical protein
*PB_transcript_17674*	CPP	Inorganic ion transport and metabolism	hypothetical protein
*PB_transcript_142867*	AUX/IAA	Transcription	auxin-responsive protein IAA13
*PB_transcript_128934*	STE	Signal transduction mechanisms	Mitogen-activated protein kinase kinase 3
*PB_transcript_24526*	CAMK OST1L	Signal transduction mechanisms	hypothetical protein
*PB_transcript_101250*	MYB	Transcription	transcriptional adapter ADA2

## Data Availability

The Illumina sequencing data used in this study has been submitted to the BioProject database of National Center for Biotechnology Information. (PRJNA1132579, https://dataview.ncbi.nlm.nih.gov/object/PRJNA1132579?reviewer=5qnhpjk3j87e8ahhm34hdefg1s accessed on 8 July 2024).

## Supplementary Materials

The following supporting information can be downloaded at: https://www.mdpi.com/article/10.3390/ijms25179653/s1.
