# Supplementary material for: Transcriptome Regulation Mechanisms Difference between Female and Male Buchloe dactyloides in Response to Drought Stress and Rehydration
Source: Int J Mol Sci. 2024 Sep 6;25(17):9653. doi: 10.3390/ijms25179653 (PMC11395050; doi:10.3390/ijms25179653)
Supplement: Supplementary file 1 [file ijms-25-09653-s001.zip › Figure.S2.pdf]

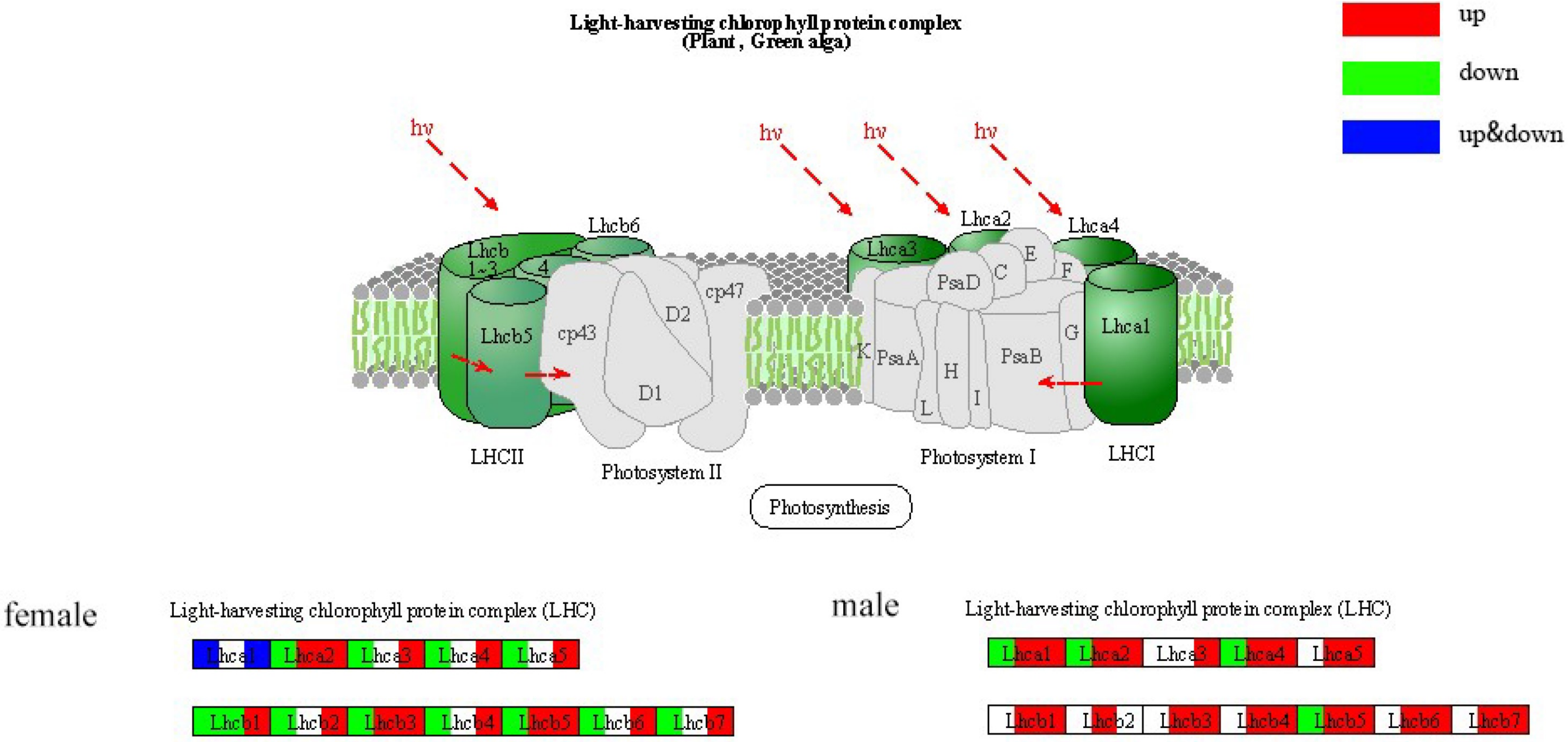

From left to right are CK vs D, D vs R, CK vs R.
